# An Unworthy Suspicion

**Published:** 1894-02

**Authors:** 


### AN UNWORTHY SUSPICION.

*Lamphear's Kansas City Medical Index*, usually a fair and impartial contemporary, surprised its Texas readers recently by repeating and emphasising a slur attempted to be cast upon the Texas Medical College.

Because Dr. T. D. Wooten, the honorable President of the Board of Regents of the Texas Uhiversity, has two sons now attending medical lectures in New York—after having attended one--perhaps two courses at Galveston—the *Index* would, by insinuation, make it appear that while Dr. Wooten thought the Medical Department of the Texas University good enough for others to graduate from, it was not good enough for his own sons; and insinuates that "there is something wrong in the management—or elsewhere,"—of that institution.

We can assure the *Index* that there is nothing "wrong," either "in the management—or elsewhere" in the Medical Department of the great University of Texas; but on the contrary, it is flourishing like a green bay tree, and everything is lovely. There are over a hundred more students in attendance this year than there were last year; and the facilities for instruction in every department are complete, and the instruction thorough, the curriculum high, and three (graded) courses are required to graduate. The greatest harmony prevails between the Faculty and the Board of Regents and between the members of the Faculty. The professors and adjunct teachers are all men of character and standing, and were selected because of their eminent fitness for their respective branches. A strict preliminary examination is required to matriculate. Funds are ample, and clinical material abundant; board is good, and can be had as reasonably as in any other city. What more could be asked or desired?

Dr. Wooten's sons are both men of legal age, and are competent and entitled to elect for themselves where they will finish their medical education.

While we do not speak from any knowledge of their reasons for going to New York, we will say that they may have though to find certain advantages there not to be had in Galveston or elsewhere; for instance, the benefit of studying with and in the office of some distinguished specialist with a view of taking a special branch. Their going to New York is no more a reflection on the Texas Medical College than would be a New York man's going to an European college a disparagement of the many colleges in New York; and it is eminently unjust, and unworthy any reputable medical journal to hold Dr. Wooten responsible for not coercing his sons, even should he not have agreed with them in their choice; or to construe their action into a reflection on the Texas College. But, "to the *jealous* mind trifles as light as air, are confirmation strong as proof from holy writ."

